# Environmental factors and potential probiotic lineages shape the active prokaryotic communities associated with healthy *Penaeus stylirostris* larvae and their rearing water

**DOI:** 10.1093/femsec/fiae156

**Published:** 2024-11-19

**Authors:** Carolane Giraud, Nelly Wabete, Célia Lemeu, Nazha Selmaoui-Folcher, Dominique Pham, Viviane Boulo, Nolwenn Callac

**Affiliations:** Ifremer, CNRS, IRD, Univ Nouvelle-Calédonie, Univ La Réunion, ENTROPIE, F-98800, Nouméa, Nouvelle-Calédonie, France; University of New Caledonia, Institut des Sciences Exactes et Appliquées (ISEA), 98800 Noumea, New Caledonia; Ifremer, CNRS, IRD, Univ Nouvelle-Calédonie, Univ La Réunion, ENTROPIE, F-98800, Nouméa, Nouvelle-Calédonie, France; Ifremer, CNRS, IRD, Univ Nouvelle-Calédonie, Univ La Réunion, ENTROPIE, F-98800, Nouméa, Nouvelle-Calédonie, France; University of New Caledonia, Institut des Sciences Exactes et Appliquées (ISEA), 98800 Noumea, New Caledonia; Ifremer, CNRS, IRD, Univ Nouvelle-Calédonie, Univ La Réunion, ENTROPIE, F-98800, Nouméa, Nouvelle-Calédonie, France; IHPE,Université de Montpellier, CNRS, Ifremer, Université de Perpignan via Domitia, 34000 Montpellier, France; Ifremer, CNRS, IRD, Univ Nouvelle-Calédonie, Univ La Réunion, ENTROPIE, F-98800, Nouméa, Nouvelle-Calédonie, France

**Keywords:** active bacteriota, commensal microorganisms, environmental factors, probiotics, shrimp larval rearing

## Abstract

Microbial dysbiosis is hypothesized to cause larval mass mortalities in New Caledonian shrimp hatcheries. In order to confirm this hypothesis and allow further microbial comparisons, we studied the active prokaryotic communities of healthy *Penaeus stylirostris* larvae and their surrounding environment during the first 10 days of larval rearing. Using daily nutrient concentration quantitative analyses and spectrophotometric organic matter analyses, we highlighted a global eutrophication of the rearing environment. We also evidenced drastic bacterial community modifications in the water and the larvae samples using Illumina HiSeq sequencing of the V4 region of the 16S rRNA gene. We confirmed that *Alteromonadales, Rhodobacterales, Flavobacteriales, Oceanospirillales*, and *Vibrionales* members formed the core bacteriota of shrimp larvae. We also identified, in the water and the larvae samples, several potential probiotic bacterial strains that could lead to rethink probiotic use in aquaculture (*AEGEAN 169 marine group, OM27 clade, Ruegeria, Leisingera, Pseudoalteromonas*, and *Roseobacter*). Finally, investigating the existing correlations between the environmental factors and the major bacterial taxa of the water and the larvae samples, we suggested that deterministic and stochastic processes were involved in the assembly of prokaryotic communities during the larval rearing of *P. stylirostris*. Overall, our results showed that drastic changes mostly occurred during the zoea stages suggesting that this larval phase is crucial during shrimp larval development.

## Teaser

In order to help understand the microbial assembly pattern occurring during shrimp larval rearing in an aquaculture system, we present a study focusing on both the active bacterial communities associated with shrimp larvae and their rearing water.

## Background

In animals, microbial symbionts colonize various types of tissues and produce many metabolites influencing the digestion, the growth, and the immune system of the host as well as other key physiological processes (Nayak 2010, Huttenhower et al. 2012, Ursell et al. 2012, Moloney et al. 2014, Ikeda-Ohtsubo et al. 2018). The establishment and the modulation of the microbiota of a given host often result from complex interactions between environmental factors and vertically transmitted microbial communities as well as host selection pressure and genetic background (Ikeda-Ohtsubo et al. 2018, Sun and Xu 2021). For example, such processes have been suggested to modulate the commensal microorganisms associated with the early life stages of Pacific blue shrimp *Penaeus stylirostris* larvae (Giraud et al. 2021, 2022, Callac et al. 2024). These very specific interactions result in hosts harboring a very well-adapted microbiota (Wippler et al. 2016). Under biotic or abiotic stress, this microbiota can be disrupted by an expansion of r-strategist microorganisms and opportunistic pathogens or by a loss of beneficial microorganisms and microbial diversity leading to a microbial dysbiosis (Petersen and Round 2014, Egan and Gardiner 2016, Vadstein et al. 2018). In marine animals, such a dysbiosis has been suggested to lead to opportunistic or polymicrobial infections causing disease outbreaks in marine ecosystems (Egan and Gardiner 2016) and more particularly in aquaculture systems (Infante-Villamil et al. 2021). For example, in the Pacific white shrimp *P. vannamei*, modifications of the gut microbiota were correlated with disease severity and beneficial bacteria were associated with healthy individuals, while potential pathogenic strains were highlighted in diseased shrimps (Xiong et al. 2015, Cornejo-Granados et al. 2017). Additionally, microbial dysbiosis in the rearing water and the larvae of the Pacific blue shrimp *P. stylirostris* have been hypothesized to explain massive larval mortalities occurring in New Caledonian hatcheries (Callac et al. 2022).

In New Caledonia, the semi-intensive farming of *P. stylirostris* leads to an annual estimated production of 1500 tons and allows the employment of over 500 people. Shrimp farming is currently the island's first food-processing exporter and is therefore a very important socio-economic activity for the territory (Agence rurale NC 2021). Unfortunately, due to massive larval mortalities occurring in the hatcheries, the post-larvae production decreased by 25% between 2005 and 2019, and it is currently threatening the sustainability of shrimp farming in New Caledonia (Pham et al. 2014). Routine tests, performed by New Caledonian veterinary services whenever larval mortalities are observed in the hatcheries, have ruled out the involvement of viral infections and bacterial septicemia. Furthermore, previous studies have excluded the possibility of breeder-related issues (Pham et al. 2012, 2014). In order to confirm the potential role of dysbiosis in larval mortalities, a characterization of the microbiota associated with healthy *P. stylirostris* larvae and their rearing water seemed necessary to allow microbial comparisons between healthy and moribund rearings. Thus, we present a new study focusing on the temporal evolution of the prokaryotic communities associated with healthy *P. stylirostris* larvae as well as with their surrounding environment during the first ten days of larval rearing. Using high-throughput Illumina sequencing of the active microbiota, we were able to identify the major bacterial orders daily associated with the water and the larvae samples. We further investigated these bacterial communities using linear discriminant analyses (LDA) effect size (LEfSe) according to the rearing day and the larval developmental stage. We highlighted several potential probiotic strains with varying abundances throughout the experiment leading to rethink probiotic use during the larval rearing of marine species (i.e. a unique bacterial strain regularly inoculated throughout the rearing process). Finally, we investigated the correlations existing between the highlighted microbial biomarkers and the physicochemical parameters of the rearing water. These results show that deterministic and stochastic processes may be involved in the assembly of the prokaryotic communities associated with *P. stylirostris* larvae and their rearing water.

## Materials and methods

### Study design

**Figure 1.**
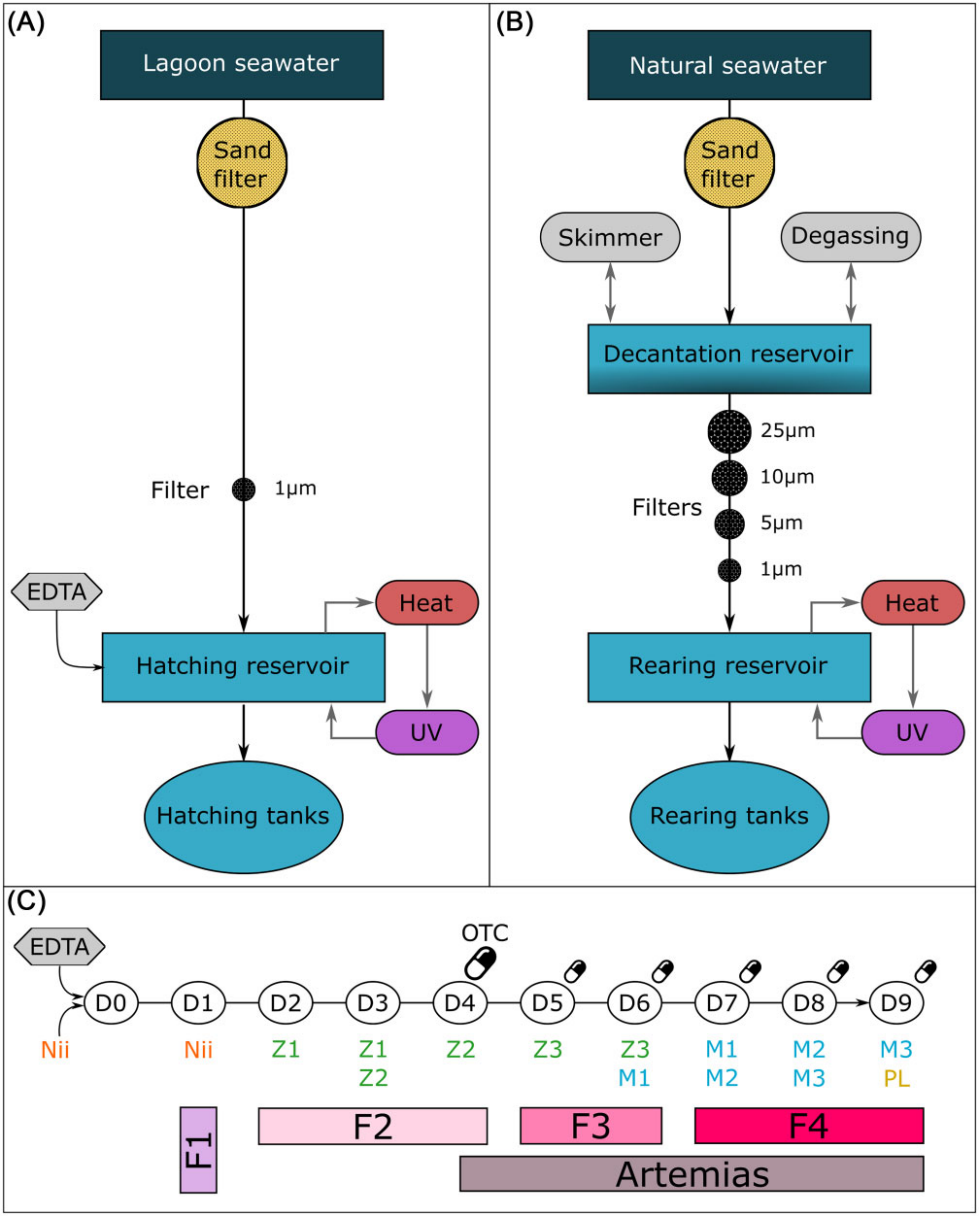
Water treatments and larval rearing process. Natural lagoon seawater was pumped, treated, and used to fill (A) the hatching tanks and (B) the rearing tanks. (C) Larval rearing cycles started when nauplii (Nii) were added to the rearing tanks after ethylenediaminetetraacetic acid (EDTA) addition on Day 0 (D0). On Day 4 (D4), 70 ppm of oxytetracycline (OTC) were added to the rearing tanks. Daily OTC doses were reduced to 17 ppm from Day 5 (D5) to Day 9 (D9). On Day 1 (D1), larvae were at nauplius stage (Nii) and were fed with a first type of microparticle (F1). From Day 2 (D2) to Day 4 (D4), larvae were at zoea 1 (Z1) and zoea 2 (Z2) stages and were fed with a second type of microparticle (F2). On Days 5 and 6 (D5 and D6), larvae were at stages zoea 3 (Z3) and mysis 1 (M1) and were fed with a third type of microparticle (F3). From Day 7 (D7) to Day 9 (D9), larvae were at mysis 1, 2, 3 (M1, M2, M3) and post-larvae (PL) stage and were fed with a fourth type of microparticle (F4). Microparticles were supplemented with frozen *Artemias* sp. from Day 4 (D4) to Day 9 (D9).

All samples were collected during an experiment conducted in August 2021 in a New Caledonian private shrimp hatchery. Prior to larval rearing, mature male and female breeders were used to perform artificial inseminations, as described by Pham et al. (2012); and 250 l hatching tanks were filled with treated natural seawater from the lagoon. Briefly, lagoon seawater (LS) was pumped through a sand filter and a 1 µm pore size filter and stocked in a hatching reservoir equipped with heating and UV cycling systems. Before filling the hatching tanks, ethylenediaminetetraacetic acid (EDTA) was added to the hatching water reservoir (15 ppm) (Fig. 1A). After artificial inseminations, inseminated females were placed in the hatching tanks at a rate of 1 individual per tank and were left to spawn in the dark. After spawning, females were retrieved while eggs were left in the hatching tanks. The next day, after hatching, the released nauplii (first larval stage) were pooled, abundantly rinsed, and disinfected with Virkon^®^ (40 µg.l^−1^) during 20 min. On the same day, 10 m^3^ rearing tanks were filled with treated natural seawater from the lagoon. Briefly, LS was pumped the day before larval rearing through a sand filter and sent to a decantation reservoir (DR) equipped with a skimmer and a degassing system. Water from the DR was then filtered through a series of 25, 10, 5, and 1 µm pore size filters and stocked in a rearing reservoir (RR) equipped with heating and UV cycling systems (Fig. 1B). Before nauplii were added at a rate of 180 individuals per liter to the rearing tanks (Day 0 of the experiment), EDTA was added (15 ppm). Progressive bubbling was implemented during the whole rearing process, salinity was kept at 30 ppt (± 1 ppt), and no water renewal was performed. From Day 4 (D4) to the end of the experiment (D9), oxytetracycline (OTC) was daily added to the rearing tanks. The first antibiotic dose was added at 70 ppm and was reduced to 17 ppm the following days. From D2 to D9, larvae were fed with different sources of microparticles according to their larval stage. Diet was supplemented with frozen *Artemias* sp. larvae from D4 to the end of the experiment (Fig. 1C). All feed sources were given every 2 h from 4 a.m. to 10 p.m. In all rearing tanks, to ensure larval transitioning from nauplius to zoea stages, temperature was increased from D0 to D2 (27–30°C) and remained stable (29.9 ± 0.4°C) from D3 to D9.

During this experiment, two larval rearing cycles were considered and were named A and B. These rearing cycles were conducted using the same reservoir water but larvae were obtained from artificial inseminations performed using different breeders on two successive days. For each rearing cycle, triplicates were performed (A1, A2, A3, B1, B2, B3). A 250-l tank filled with the same water as the rearing tanks and with no larvae or food addition was considered to be the control of our experiment (Ctrl).

### Sample collection

As larval rearing cycles A and B were conducted using the same reservoir water, unique samples of LS, DR, and RR were collected prior to the beginning of the experiment (Fig. 1B). For each sample, 1 l of seawater was collected using a 2 l plastic sampling bottle. For each larval rearing cycle, on the day the hatching tanks were filled, 1 l of water was collected from the hatching reservoir (named HR_A and HR_B). On the same day, after spawning, around a hundred eggs were sampled from the hatching tanks in 2 ml sterile microtubes using a 100 µm pore size net and sterilized tweezers. The next day, 1 l of water was collected from all the rearing tanks after EDTA addition and before nauplii addition (W_D0). On the same day, around a hundred nauplii were sampled after the disinfection process and before addition to the rearing tanks (L_D0). From D1 to D9, prior to the first feeding of the day, larvae (L) and water (W) were sampled daily from all the rearing tanks. Around 100 larvae were collected in sterile 2 ml microtubes using a 120 µm pore size net and sterilized tweezers. In order to sample 1 l of rearing water with no larvae, a 120 µm filtering device and a 2 l plastic sampling bottle were used. Additionally, water samples were daily collected from the control tank at the same sampling time as the rearing tanks. Every food type was sampled once during the experiment in 2 ml sterile microtubes using sterilized tweezers (F1, F2, F3, F4, *Artemias* sp.).

All water samples were filtered using 0.2 µm pore size filters (S-PAK membrane filter, Millipore). Filters as well as egg, larvae, and food samples were stored at −80°C until further RNA extractions. Filtered water was used to fill 40 ml sterile polyethylene vials, which were stored at −20°C for further total ammonia nitrogen (TAN) and Soluble Reactive Phosphorus (SRP) quantitative analyses. Filtered water was also used to fill 50 ml pre-combusted (8 h, 450°C) glass bottles in order to perform colored dissolved organic matter (CDOM) spectrophotometric analyses within hours after the sample collection.

### Zootechnical parameters

For each rearing tank, larvae contained in 1 l of rearing water were daily observed under a binocular magnifying glass in order to determine larval stages and survival rates.

In order to evaluate larval health status of the rearing cycles, larval survival rates (LSR) and larval stage indexes (LSI) were calculated and compared to reference values (Fig. S1). These references were estimated by averaging LSR and LSI values for each rearing day using 10 years of successful rearing data (Pham, comm. pers.). The LSR was determined using the following equation: (*n* / T) x 100, where n is the number of counted living larvae in the rearing tank and T the number of larvae added to the rearing tank on D0.

The larval stage index (LSI) was determined using a modified equation of Maddox and Manzi (1976): (0*Nii + 1*Z1 + 2*Z2 + 3*Z3 + 4*M1 + 5*M2 + 6*M3 + 7*PL)/N, where Nii corresponds to the number of larvae observed at nauplius stage, and Z1, Z2, and Z3 indicate the number of larvae respectively observed at stages zoea 1, 2, and 3. In the same way, M1, M2, and M3 correspond to larval stages mysis 1, 2, and 3. PL stands for the number of observed post-larvae. Finally, N indicates the total number of observed larvae on the rearing day of interest.

In addition to these survival and stage indexes, daily larval observations were conducted to assess larval health indicators, including feeding behavior, visible signs of disease, and intestinal content (Zheng et al. 2017).

### Water quality analyses

For all water samples, temperature and pH were daily measured using the multiparameter probe pH/Cond 3320 (Mettler Toledo).

Concentrations of total ammonia nitrogen (TAN) and SRP were respectively measured using the methods described by Holmes et al. (1999) and Murphy and Riley (1962).

CDOM analyses were performed by determining the absorption spectra of the water samples from 200 to 700 nm using a 10 cm quartz tank and a SHIMADZU UV-1800 spectrophotometer (Helms et al. 2009). Milli-Q water was considered to be the reference of our analysis. The absorption coefficient at 325 nm (aλ_325 nm_) was used as a proxy of the CDOM concentration (Sadeghi-Nassaj et al. 2018) and was determined using the following equation: (2.303 * a)/l, where 2.303 is the logarithm transformation factor, a is the measured absorption at 325 nm, and l is the length of the quartz tank (10 cm). The slope ratios of the absorption spectra from 275 to 295 nm (S_275-295_) and from 350 to 400 nm (S_350-400_) were determined in order to calculate the total slope ratio (SR) using the following equation: S_275-295_/S_350-400_.

### RNA extractions and sequencing

RNA extractions were performed using the RNeasy PowerWater kit (Qiagen) for the water samples and the RNeasy minikit (Qiagen) for the egg, the larvae, and the food samples. All RNAs were reverse-transcripted into complementary DNAs (cDNAs). First, 200 ng of RNAs were added to a reaction mix (buffer 5X, dNTP 10 mM, random hexamers 50 µM, reverse transcriptase M-MLV (PROMEGA) 200 u.µl^−1^, RNAse/DNAse free water). Then, reverse transcription was performed in a thermocycler during 10 min at 25°C, 2 h at 42°C, and 5 min at 85°C. Finally, reverse transcription was completed using the Second Strand cDNA Synthesis kit (ThermoFisher).

Obtained cDNAs were sent to MrDNA (Shallowwater, TX, United States) to amplify and sequence the V4 region of 16S rRNA gene using the 515F/806R primers (Caporaso et al. 2011). Following this step, HiSeq Illumina sequencing was performed using a 2 × 150 pb paired-end run and an average sequencing depth of 50 k raw reads per sample. Raw sequences obtained from the HiSeq Illumina sequencing were treated using the DADA2 package (Callahan et al. 2016) implemented in the R software (v4.1.2) in RStudio (2022.07.2 build 576) (RStudio Team 2020) as described in Giraud et al. (2021). Prior to bacteriota analysis, sequences affiliated to Eukaryotas, Chloroplasts, and Mitochondrias were removed from the ASV table. A total of 68 490 986 reads were found and 13 291 ASVs were evidenced. Minimum library size was 117 730 reads and maximum library size was 1474 219 reads. As a consequence, the final ASV table was normalized using the CPM method implemented in the normalize function of the phyloseq package in RStudio (McMurdie and Holmes 2013).

The datasets generated and analyzed during this study are available in the NCBI SRA repository (BioProject PRJNA736535, SRA SRR21099695 to SRR21099837).

### Downstream analysis

All downstream analysis was performed using the R software (v4.1.2) implemented in RStudio (2022.07.2 build 576) (RStudio Team 2020). Using the ggplot2 package (Wickham 2016), a heatmap was built to visualize zootechnical and physicochemical parameters. In order to reduce dimensions and to simplify visual representations of these parameters, a standardized principal component analysis (PCA) was performed using the dudi.pca function of the ade4 package (Thioulouse et al. 2018). Influences of rearing days and larval stages on environmental factors were tested by performing a PERMANOVA using the adonis2 function of the vegan package (Oksanen et al. 2020).

Microbial analyses were conducted using the phyloseq package (McMurdie and Holmes 2013). Alpha diversity indexes were determined using the plot_richness function and Principal Coordinates Analysis (PCoA) ordinations were built using the ordinate and plot_ordination functions. A dendrogram based on a Bray-Curtis dissimilarity matrix and the Ward method was also constructed using the vegan package (Oksanen et al. 2020). Linear discriminant analyses (LDA) effect size (LEfSe) (Segata et al. 2011) were performed using the microbiomeMarker package (Cao et al. 2022). Relative abundances of the ASVs highlighted by the LEfSe with a threshold set at 4, were visualized by constructing scatterplots using the ggplot2 package (Wickham 2016). The microeco package (Liu et al. 2021) was used to build correlograms between the physicochemical parameters and the ASVs highlighted by the LEfSe.

## Results

### Zootechnical and physicochemical parameters

**Figure 2.**
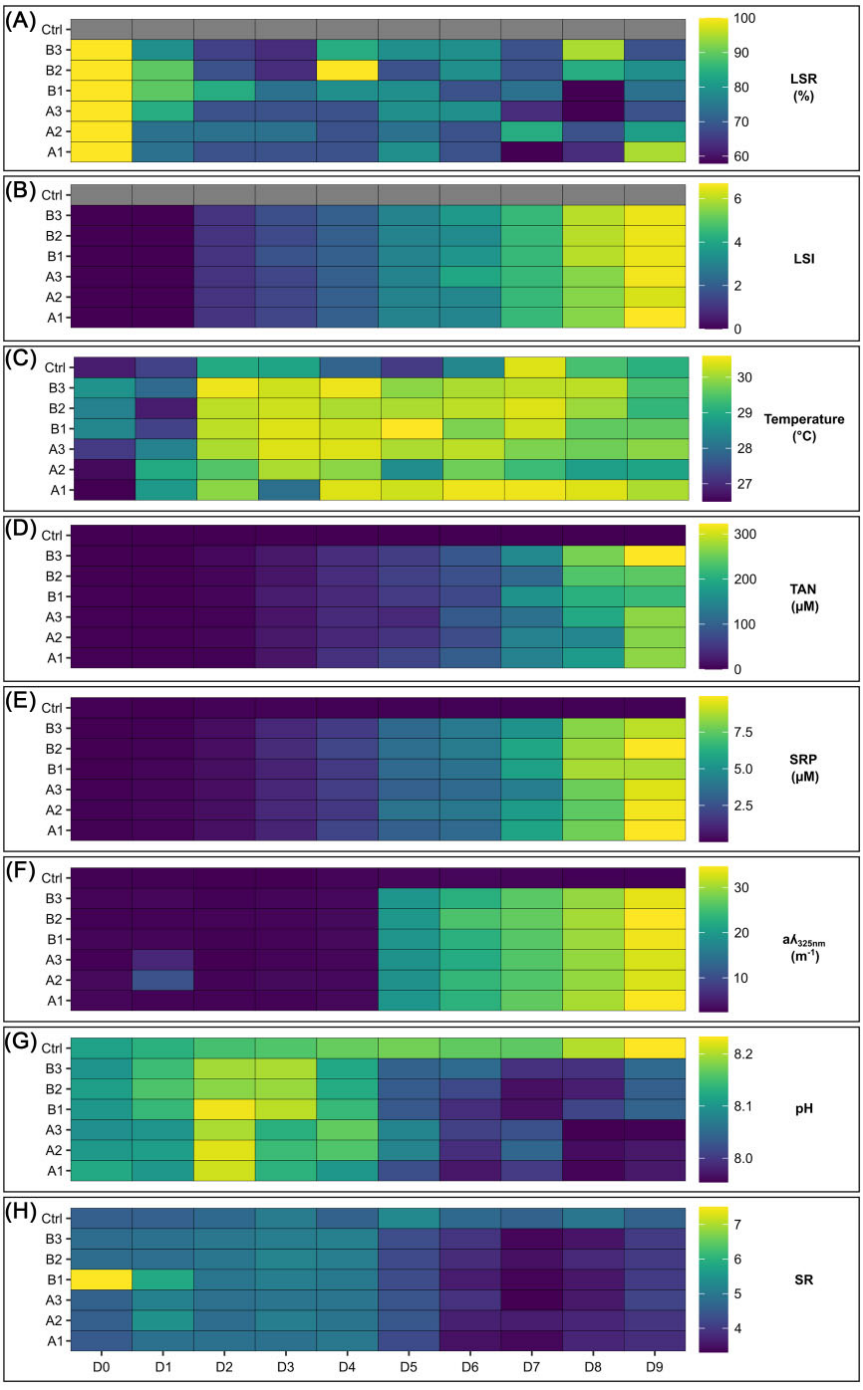
Heatmap of all zootechnical and physicochemical parameters in the rearing and the control tanks. (A) LSR and (B) LSI in all the rearing tanks (larval rearing cycle A: A1, A2, A3, larval rearing cycle B: B1, B2, B3) from Day 0 (D0) to Day 9 (D9). As no larvae were added to the control (Ctrl) tank, no LSR or LSI were calculated. (C) Temperature, (D) total ammonia nitrogen (TAN) concentration, (E) SRP concentration, (F) CDOM concentration (aλ_325 nm_), (G) pH, and (H) SR values in the rearing and the control tanks from D0 to D9. .

Zootechnical parameters were only considered in the rearing tanks, as larvae were not added to the control tank. LSR decreased in all rearing tanks and the lowest observed value on the final day of the experiment was 69% (A3 and B3 tanks) (Fig. 2A). As larvae grew, LSI increased in all rearing tanks (Fig. 2B), and, as expected, all observed larvae were transitioning to post-larvae stage (LSI = 7) on Day 9 (D9). All zootechnical parameters were homogeneous between the 2 larval rearing cycles (A and B) and mimicked reference values (Fig. S1). Thus, both rearing cycles were considered healthy.

In all rearing tanks, temperature increased from D0 to D2 and remained stable from D3 to D9 (Fig. 2C). Ammonia (TAN) and phosphorus (SRP) concentrations increased progressively during both larval rearing cycles compared to the control tank (Fig. 2D and E). Indeed, TAN and SRP concentrations were null or near 0 µM in the rearing tanks at the beginning of the experiment and respectively reached 322 and 9.9 µM on D9, while values remained low in the control tank. CDOM concentration (aλ_325 nm_) values were also significantly different as they increased in all rearing tanks compared to the control tank (Fig. 2F). More precisely, aλ_325 nm_ values shifted between D4 and D5 as they varied from 2 to 5 m^−1^ from D0 to D4 and from 19 to 35 m^−1^ from D5 to D9. On the contrary, pH (Fig. 2G) and SR (Fig. 2H) values decreased from D5 to the end of the experiment. From D0 to D4, in the rearing tanks, pH values increased up to 8.2 and then decreased to 8 by the end of the experiment, while pH was never <8.1 in the control tank. SR values remained stable in the control tank while they equaled 5 from D0 to D4 and decreased to 3.8 from D5 to D9 in the rearing tanks.

**Figure 3.**
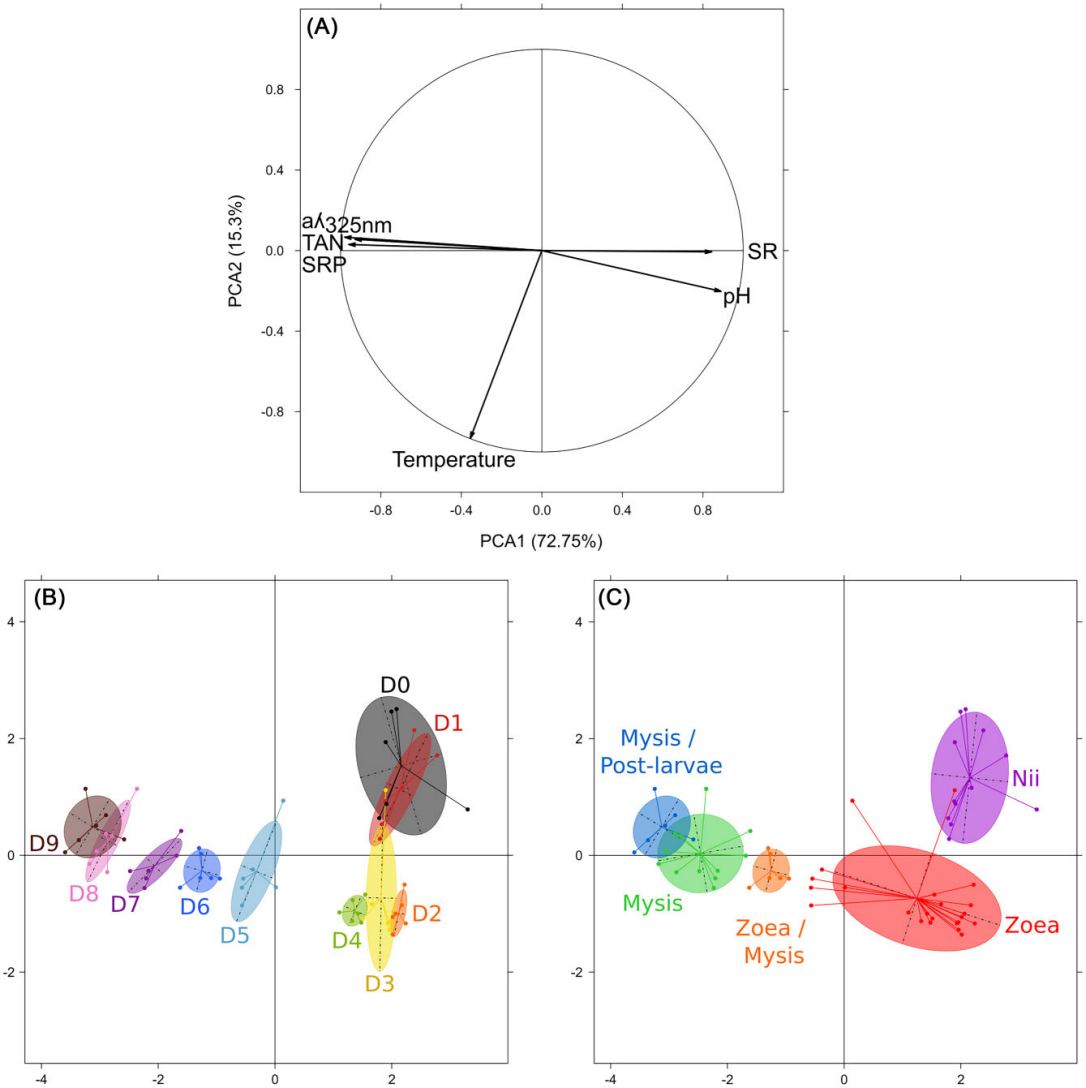
Standardized Principal Component Analysis (PCA) of the physicochemical parameters in the rearing water of all the samples from Day 0 (D0) to Day 9 (D9). (A) Correlation circle of the physicochemical parameters measured in the rearing tanks throughout the whole rearing cycles. SRP concentration, total ammonia nitrogen (TAN) concentration, CDOM concentration (aλ_325_ _nm_), SR, pH and temperature, values were considered. (B and C) Visual representation of the samples according to their physicochemical composition. (B) Samples are clustered by rearing day, from Day 0 (D0) to Day 9 (D9). (C) Samples are clustered by larval stage, from nauplii (Nii) to post-larvae stage.

The correlation circle obtained from the standardized PCA (Fig. 3A) showed that aλ_325 nm_, TAN, and SRP variables as well as pH and SR values were respectively positively correlated. The first two principal components (PCA1 and PCA2), which explained 87% of the total inertia, seemed to be driven by a pH and organic matter gradient as well as by a temperature gradient.

Samples collected at D0 and D1, when larvae were at nauplius (Nii) stage, were characterized by low temperature values as well as low TAN, SRP, and CDOM (aλ_325 nm_) concentrations and by high pH and SR values (Fig. 3B and C). When larvae grew to zoea stage, from D2 (Z1) to D4 (Z2), samples were characterized by higher temperature values (Fig. 3B and C). Finally, during the late zoea (Z3) and all the mysis stages, from D5 to D9, physicochemical composition of the samples shifted and was characterized by decreasing SR and pH values and by increasing TAN, SRP, and CDOM concentrations (Fig. 3B and C). Thus, it appeared that physicochemical composition of the rearing water samples varied according to the rearing day and the larval stage (PERMANOVA, *P* = 0.001).

### Microbial compositions of all the samples

**Figure 4.**
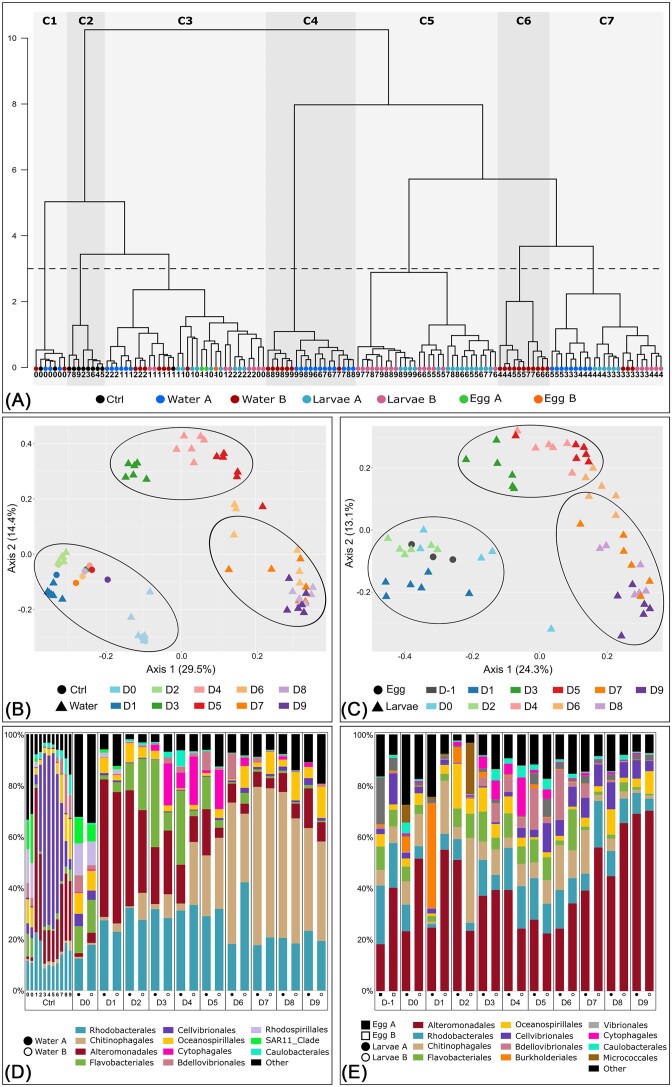
Clustering and global microbial composition of the water and the larvae samples. Two larval rearing cycles were analyzed during the experiment and are indicated as A and B. (A) Hierarchical clustering based on a Bray–Curtis dissimilarity matrix and the Ward method groups the water, the larvae, the egg, and the control (Ctrl) samples in 7 clusters (C1–C7) when considering a threshold of 3 represented by the black dotted line. Each sample type is represented by a colored circle and rearing days are noted under the circles. (B) Ordination of the control (Ctrl) and the water samples based on the PCoA method and a Bray–Curtis dissimilarity matrix. (C) Ordination of the egg and the larvae samples based on the PCoA method and a Bray–Curtis dissimilarity matrix. In Fig. 4B and C, clusters are represented by black circles. (E) Histogram of the 11 bacterial orders with a relative abundance higher than 1% in the control and the water samples. (D) Histogram of the 12 bacterial orders with a total relative abundance higher than 1% in the egg and the larvae samples. In Fig. 4D and E, all orders with a relative abundance lower than 1% are summed in the “Other” category. In Fig. 4B–E, all rearing days are indicated by the letter D followed by the number of the rearing day (from 0 to 9).

Hierarchical clustering of all samples highlighted 7 clusters (C1 to C7 in Fig. 4A). All water and larvae samples collected from D0 to D2 as well as all egg and control samples were distributed in clusters C1 to C3, belonging to the same larger cluster, whereas clusters C4 to C7 formed a large group gathering all water and larvae samples from D3 to D9 (Fig. 4A). Thus, prokaryotic compositions of the samples changed after the second day of rearing. Additionally, water and larvae samples were mixed in the clusters associated to the first rearing days (D0 to D2), while they were clustered in separate groups from D3 to D9. Furthermore, during that period, another bacterial composition shift seemed to occur between D4 and D6 in all samples. This was reinforced by ordination analysis, which showed three clusters gathering samples collected from D0 to D2 (nauplius and zoea 1 stages), from D3 to D5 (zoea 2 and zoea 3 stages), and from D7 to D9 (mysis and post-larval stages) (clusters are circled in Fig. 4B and C). Thus, as for the physicochemical parameters, it appeared that bacterial compositions of water and larvae samples changed according to the rearing day and, thus, to the developmental stage of the larvae in the rearing tanks. Histograms allowed to highlight major bacterial orders associated with water and larvae samples and displayed major shifts in bacterial diversity (Fig. 4D and E).

### Enriched ASVs in the water and larvae samples according to the rearing day and larval stage

**Figure 5.**
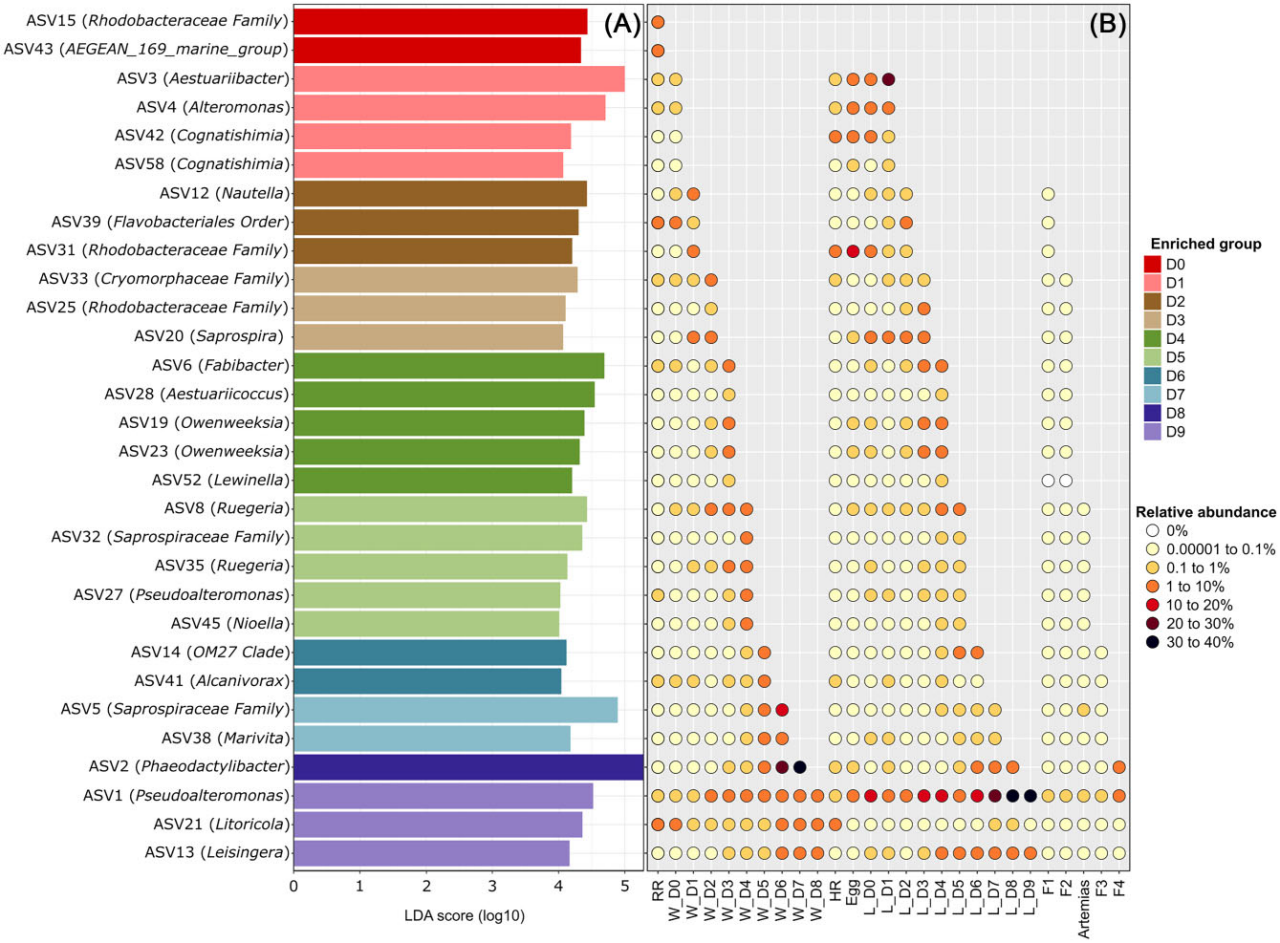
Differentially abundant ASVs in all the rearing water samples (larval rearing cycles A and B) according to their rearing day and relative abundance of these ASVs in all the samples. (A) Linear discriminant analysis (LDA) effect size (LEfSe) showing ASVs significantly more abundant in the rearing water samples according to their rearing day, from Day 0 (D0) to Day 9 (D9). For each ASV, the lowest phylogenetic level reached for affiliation is specified in brackets. (B) Relative abundance of the ASVs highlighted by the LEfSe in all the samples prior to the rearing day during which they had been identified. For all the samples, the mean relative abundance per sample type is represented. The water and the larvae samples are respectively designated by W and L. The relative abundances of the ASVs in the LS, the DR, and the rearing reservoir are averaged in RR. The samples collected from the hatching reservoir for the larval rearing cycles A and B are averaged in HR. Food types, microparticle mixes, and *Artemias* sp. larvae, are respectively represented by Fx (with x from 1 to 4) and *Artemias*.

The first LEfSe was conducted to analyze enriched ASVs in the water samples according to their rearing day (Fig. 5A). We then analyzed the relative abundances of the highlighted ASVs in all the samples in order to observe if they were present since the beginning of the rearing process in the water and if they were also highlighted in the larvae and the food sources up to the considered rearing day (Fig. 5B). A total of 30 ASVs were highlighted (Fig. 5A) and were all found in the water reservoirs, the larvae, and the rearing water samples. Their relative abundance increased in these samples throughout the rearing days. All the ASVs had a relative abundance lower than 1% in the food samples except for ASVs 1, 2, and 52 (Fig. 5B).

**Figure 6.**
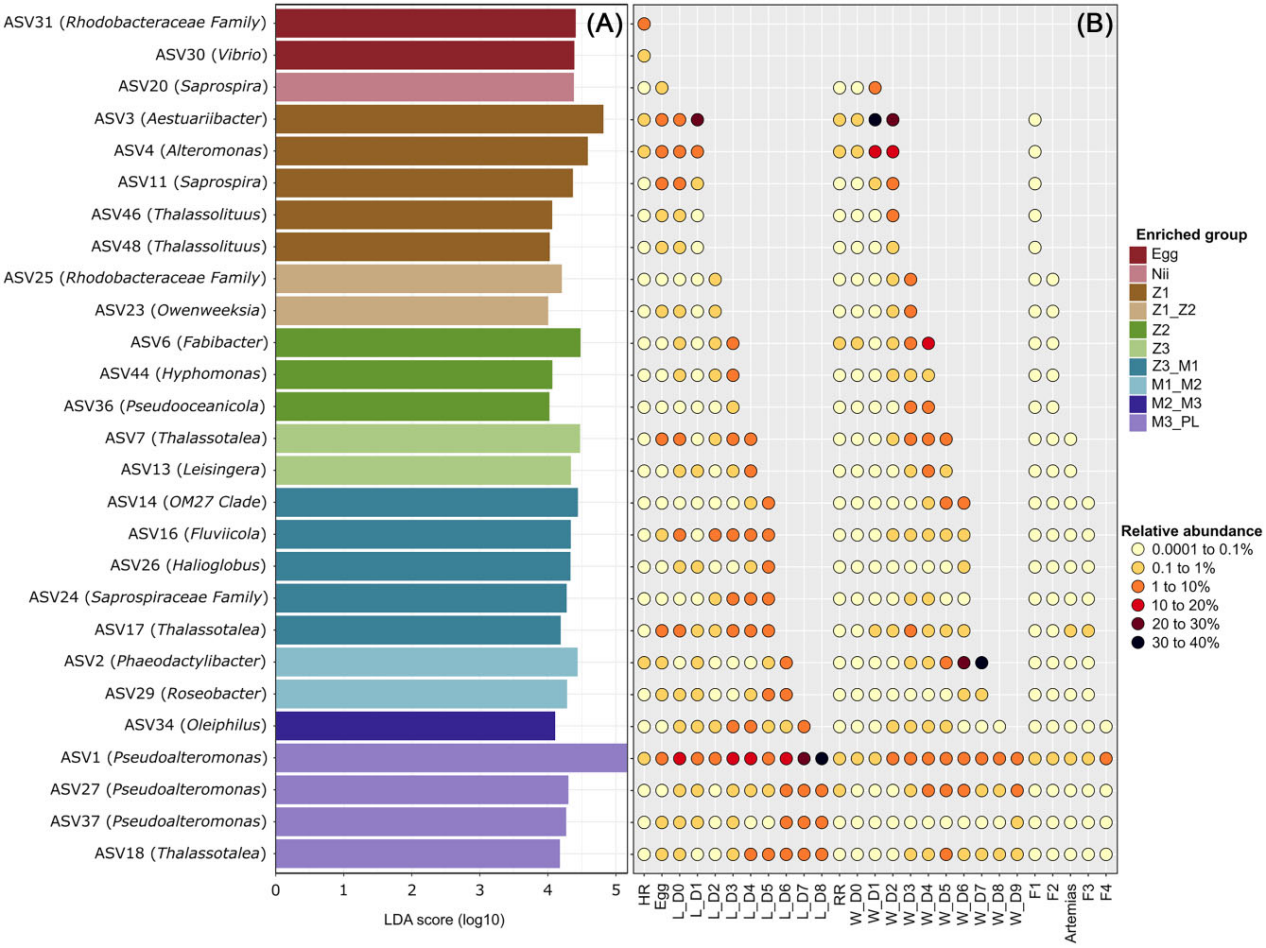
Differentially abundant ASVs in all the egg and the larvae samples (larval rearing cycles A and B) according to their larval stage and relative abundance of these ASVs in all the samples. (A) Linear Discriminant Analysis (LDA) Effect Size (LEfSe) showing ASVs significantly more abundant in the egg and the larvae samples according to their larval stage: egg, nauplii (nii), zoea 1 (Z1), zoea 2 (Z2), zoea 3 (Z3), mysis 1 (M1), mysis 2 (M2), mysis 3 (M3), and post-larvae (PL). For each ASV, the lowest phylogenetic level reached for affiliation is specified in brackets. (B) Relative abundance of the ASVs highlighted by the LEfSe in all the samples prior to the larval stage in which they had been identified. For all the samples, the mean relative abundance per sample type is represented. The larvae and the water samples are respectively designated by L and W. The samples collected from the hatching reservoir for the larval rearing cycles A and B are averaged in HR. The relative abundances of the ASVs in the LS, the DR, and the rearing reservoir are averaged in RR. Food types, microparticle mixes, and *Artemias* sp. larvae, are respectively represented by Fx (with x from 1 to 4) and *Artemias*.

An LEfSe was also conducted considering the differentially abundant ASVs in the egg and the larvae samples according to their developmental stage (Fig. 6). As for the first LEfSe, we analyzed the relative abundances of these ASVs in all the samples in order to observe if they were present since the beginning of the rearing process in the larvae and if they were also highlighted in the water and the food sources up to the considered rearing day. A total of 25 ASVs were highlighted and were all found in the reservoir samples and in all larvae and water samples collected prior to the considered sampling time. Relative abundances in these samples increased throughout the rearing cycle. The ASVs were also found in the food samples but almost always had a relative abundance inferior to 0.1%. Thus, we highlighted bacterial biomarkers up to the genus level, when possible, of the rearing day and the larval stage for the water and the larvae samples, and all these prokaryotic strains were present in the water reservoirs since the beginning of the experiment.

### Correlations between bacterial biomarkers and physicochemical parameters

**Figure 7.**
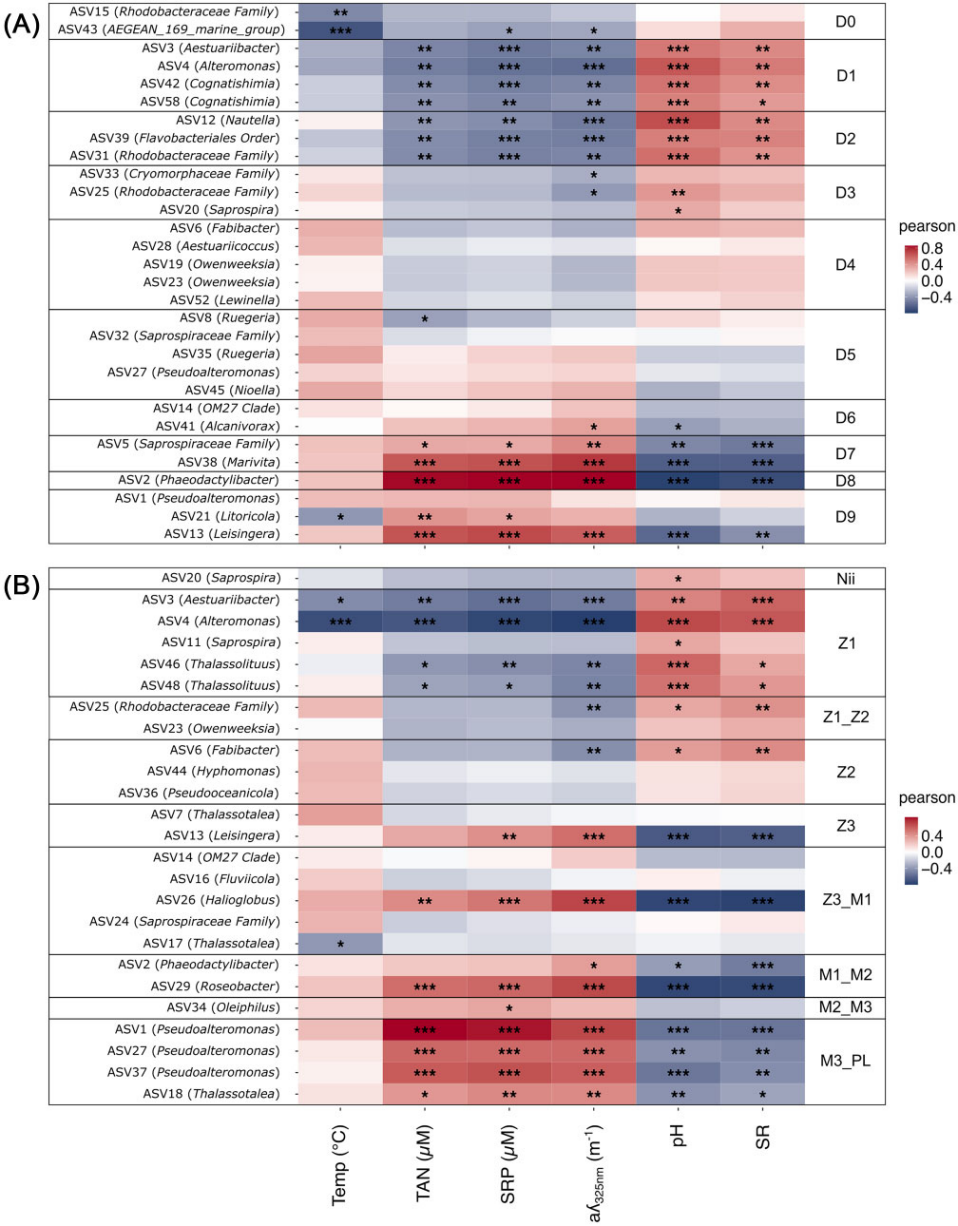
Pearson correlations between the microbial biomarkers and the physicochemical parameters of the rearing water. Correlations between the physicochemical parameters of the rearing water: temperature (Temp), total ammonia nitrogen (TAN) concentration, SRP concentration, CDOM concentration (aλ_325 nm_), pH, and SR values were considered and the microbial biomarkers highlighted by the LEfSe analyses in (A) the water samples and (B) the larvae samples. Rearing days are represented by the letter D followed by the considered rearing day (from 0 to 9). Larval developmental stages are designated as follows: nauplii (Nii), zoea 1 (Z1), zoea 2 (Z2), zoea 3 (Z3), mysis 1 (M1), mysis 2 (M2), mysis 3 (M3), and post-larvae (PL). Significant results are designated by an asterisk symbol: *P*-values comprised from 0.01 to 0.05 are represented by *, *P*-values comprised from 0.001 to 0.009 by **, and *P*-values below 0.0009 by ***.

In the water samples, 19 out of the 30 ASVs highlighted by the LEfSe were significantly positively or negatively correlated with at least one environmental factor (Fig. 7A). Temperature was negatively correlated with members of the *Rhodobacteraceae* family and the *AEGEAN 169 marine group*, which were both enriched on D0, as well as with an ASV affiliated to *Litoricola* on D9. All the ASVs enriched in the water samples on D1 and D2 were negatively correlated with ammonia, phosphorus, and CDOM concentrations (TAN, SRP, and aλ_325 nm_), while they were positively correlated with pH and SR. ASVs which were highlighted by the LEfSe from D7 to D9 showed an opposite profile. A total of 15 ASVs were enriched from D3 to D6 and only 5 of them were correlated with physicochemical parameters.

In the larvae samples, 25 ASVs were highlighted by the LEfSe analysis and 18 of them were correlated with the physicochemical parameters (Fig. 7B). ASVs highlighted at zoea 1 stage were negatively correlated with TAN, SRP, and CDOM concentration (aλ_325 nm_) but positively correlated with pH and SR. Opposite trends were highlighted for ASVs enriched during the zoea 3 stage and the transitioning phases between mysis 1 and 2 as well as between mysis 3 and post-larvae.

## Discussion

### Temporal evolution of physicochemical parameters in the rearing water

Ammonia (TAN) and phosphorus (SRP) concentrations progressively increased (Fig. 2D and E) suggesting an eutrophication of the rearing water throughout the experiment. These nutrients are essential to the development of most organisms but can be harmful if present in excess. Indeed, ammonia and phosphorus pollution can disturb growth, metamorphosis, and survival of several *Penaeidae* shrimp species at post-larvae, juvenile, and adult stages (Chen and Kou 1992, Xia et al. 2004, Magallon Barajas et al. 2006, Zhao et al. 2020). In our study, according to LSR (Fig. 2A), LSI (Fig. 2B), and daily visual inspections, all larval rearings were considered healthy. To our knowledge, these three methods are the only ones that have been used in studies focusing on healthy shrimp larval bacteriota (Zheng et al. 2017, Wang et al. 2020a, Callac et al. 2022, Callac et al. 2023). As no water renewal was performed during rearing cycles, the observed eutrophication could be explained by the accumulation of uneaten food, feces, and larval molts as previously described during the larval rearing of *P. stylirostris* (Callac et al. 2022). Overall, the environmental conditions drastically changed between the beginning and the end of the rearing process through a temperature increase (Fig. 2C), a global eutrophication of the water (Fig. 2D and E), and a major environmental shift occurring between D4 and D5 (Fig. 2F–H) when larvae were transitioning from zoea 2 to zoea 3 (Fig. 3). As modifications of the rearing environment can be linked to microbial dynamics in the water and in the host (Sehnal et al. 2021), we investigated if the prokaryotic communities associated with the water and the larvae samples also exhibited major modifications.

### Core bacteriota

All water and larvae samples exhibited large proportions of *Rhodobacterales* and *Alteromonadales* throughout the experiment (Fig. 4D and E). This was confirmed by the LEfSe analysis, as nearly half of the identified ASVs were affiliated to these bacterial orders (Figs 5A and 6A). *Rhodobacterales* are oligotrophic and metabolically diverse microorganisms colonizing various ecological niches within the marine environment (Garrity et al. 2015a, Haggerty et al. 2017, Simon et al. 2017). As a result, they can represent up to 30% of the total marine bacterial communities in surface seawaters, where they are essential to biogeochemical cycling (Simon et al. 2017). Like *Rhodobacterales, Alteromonadales* are able to produce many different metabolites allowing them to adapt to various ecological niches and are, thus, found in very diverse ecosystems ranging from soil to seawater (Ivanova and Mikhailov 2001). *Rhodobacterales* and *Alteromonadales* members have been identified in the rearing water and the post-larvae of the Pacific white shrimp *P. vannamei* (Hou et al. 2018, Yan et al. 2020) as well as in the intestinal microbiota of adult Chinese shrimps *Fenneropenaeus chinensis* (Liu et al. 2011, Huang et al. 2016) and in the rearing water of *P. stylirostris* larvae (Callac et al. 2022). Recently, studies focusing on the larval microbiota of *P. vannamei* and *P. stylirostris* have also highlighted microorganisms affiliated to these 2 bacterial orders (Wang et al. 2020a,b, Giraud et al. 2021, 2022, Zheng et al. 2017). Taken together, our results reinforce the hypothesis that *Rhodobacterales* and *Alteromonadales* members, originating from the rearing environment, partially form the core microbiota of several shrimp species, where they play essential roles in the physiology of their hosts during the whole life cycle. Further on, in the egg samples, prior to the beginning of the larval rearing, members of the *Flavobacteriales, Oceanospirillales*, and *Vibrionales* orders were also highlighted. This was expected as members of these bacterial orders have previously been identified in the eggs, nauplii, and breeders of *P. stylirostris*, where they formed the core bacteriota of all the considered samples (Giraud et al. 2022). Even though their abundance varied throughout the experiment, these bacterial orders were maintained in the larval bacteriota during the whole rearing process. As for *Alteromonadales* and *Rhodobacterales*, our results suggest that *Flavobacteriales, Oceanospirillales*, and *Vibrionales* members could form the core microbiota of *P. stylirostris* larvae.

### Temporal evolution of major bacterial orders in the rearing water

When the rearing tanks were filled, on D0, water samples were partly composed by *Flavobacteriales, Oceanospirillales*, and *SAR11* members. This was not surprising, as they are very common in marine environments where they play key roles in organic matter transformation and nutrient turnover (Morris et al. 2002, Bernardet 2015, Garrity et al. 2015b). *Rhodospirillales* were also highlighted in the water samples on D0. This bacterial order has previously been isolated in the stomach of healthy adult shrimps, which had been challenged with *Vibrio parahaemolyticus*, a bacteria known to induce the acute hepatopancreatic necrotic disease (Restrepo et al. 2021). Due to their antagonist activity with various *Vibrio* lineages, *Rhodospirillales* were suggested as potential probiotic strains and the authors even suggested that they could have a protective role in shrimps under rearing conditions (Restrepo et al. 2021). Furthermore, *Rhodospirillales* members have previously been identified in the rearing water of *P. stylirsotris* larvae at nauplius stage and before larval mass mortalities, which could also confirm their protective role during the early larval stages of the rearing process (Callac et al. 2022).

On the next day (D1), proportions of *Alteromonadales* largely grew and rapidly decreased in the rearing water from D2 to D5, while *Chitinophagales* abundances increased from D4 to D9. *Chitinophagales* belong to the *Bacteroidetes* phyla and are known to produce various exogeneous enzymes allowing them to degrade complex nutrient sources such as carbon polymers (Fernández-Gómez et al. 2013). As suggested by their name, they are involved in the biodegradation of chitin, a polysaccharide composing the exosqueletton of crustaceans and therefore of shrimps (McIlroy and Nielsen 2014). To our knowledge, studies focusing on the microbiota associated with various shrimp species and their rearing environment have highlighted large abundances of *Bacteroidetes* at all developmental stages but never such high proportions of *Chitinophagales* (Hou et al. 2018, Cornejo-Granados et al. 2018). As our study is the first to consider the active bacteriota associated with healthy larvae of *P. stylirostris* and their surrounding environment, these contrasting *Chitinophagales* abundances could be explained by differences in studied shrimp species and rearing environments. Furthermore, dissimilarities in the choice of DNA or RNA extraction as well as extraction technique, sequencing method, and downstream analyses could also explain these differences (Hugerth and Andersson 2017). In any case, a shift between *Alteromonadales* and *Chitinophagles* communities occurred between D3 and D5 during the zoea stage in the water samples, at the same rearing time as the previously observed physicochemical shifts (Fig. 2). Beyond larval transitioning, antibiotic as well as *Artemias* sp. larvae were added during this rearing period and could explain the observed modifications of the microbiota associated with the rearing water. However, antibiotic addition was shown to majorly impact the active rare biosphere of the surrounding water of *P. stylirostris* at the beginning of the larval rearing (Callac et al. 2022), potentially ruling out the influence of oxytetracycline (OTC) addition on the observed microbial changes in this study. Furthermore, microbial composition of *Artemias* sp. larvae was dominated by *Vibrionales* members and by an unclassified *Bacteria* (ASV86), while *Chitinophagales* only accounted for 0.2% of the total relative abundance (Fig. S3). Thus, microbial profiles were very different between this feeding source and the rearing water. Even though these observations tend to also rule out the influence of *Artemias* sp. larvae addition on the microbiota of the water, it is important to notice that food samples were collected before addition to the tanks. As a consequence, active bacterial communities associated with food sources could have increased when in contact with the rearing water or some dormant bacteria may have been activated after addition to the tanks. Finally, larval transitioning may have led to the observed increase of *Chitinophagales*. Indeed, larval molting occurs between zoea 1 and zoea 2 stage (from D2 to D4) and could have resulted in an increase of chitin in the rearing water (Wang et al. 2020a, Sathish Kumar et al. 2017). Chitin is a high molecular weight linear assembly of N-acetylglucosamine residues (Souza et al. 2011). Thus, chitin accumulation could explain the organic matter concentration increase showed by the high CDOM concentration (aλ_325 nm_) values observed on D5 (Fig. 2F). Furthermore, as high SR values involve low dissolved organic matter molecular weight, the low SR values observed during our experiment (Fig. 2G) could also be explained by the increase of high molecular weight chitin (Helms et al. 2009, Sadeghi-Nassaj et al. 2018). This hypothesis is also supported by the increase of *Cytophagales* occurring at the same time. Indeed, *Cytophagales* also belong to the *Bacteroidetes* phyla and are known to degrade biomacromolecules explaining why they are very abundant in habitats rich in organic material (Reichenbach 1992). Thus, an increase of chitin due to larval molting could also explain the increase of *Cytophagales* proportions.

When *Chitinophagales* and *Cytophagales* increased in the water samples, large proportions of *Bdellovibrionales* were also highlighted. Microorganisms affiliated to this bacterial order are *Bdellovibrio* and like organisms (BALOs) with an obligatory predatory lifestyle against Gram-negative bacteria (Snyder et al. 2002, Bratanis et al. 2020). They are considered as live antibiotics and water clean-ups as well as biocontrol agents and are currently studied for their potential use as probiotics in aquatic, poultry, and farm animals. For example, in shrimp farming, BALOs have been suggested as potential biocontrol agents to prevent *Vibrio* infections (Bratanis et al. 2020).

### Temporal evolution of major bacterial orders in the larvae

During the first 2 days of rearing, when larvae were at nauplius stage, they showed heterogeneous microbial compositions. Symbiotic microorganisms acquired from the breeders have been suggested to modulate the microbiota of several larval marine species and such a vertical transmission has been hypothesized to influence the establishment of the microbiota of *P. stylirostris* larvae (Giraud et al. 2021, 2022, Nyholm 2020). Thus, the microbial variations observed between the larval samples at nauplius stage (D0 and D1) could be explained by this influence. However, at zoea stage (from D2 to D5), bacterial compositions became more homogeneous. This was also shown by Wang et al. (2020a), who found that microbial compositions of *P. vannamei* larvae at nauplius stage were more heterogeneous than at zoea and mysis stage. The authors explained that, after the mouth opening occurring at zoea 1 stage, the larvae could have selected particular microbial communities with specific functions in order to help digestion through their immature digestive tract. Furthermore, diet and environmental microbiota have been shown to influence the gut microbiota associated with fish (Nayak 2010, Ursell et al. 2012, Sun and Xu 2021). Therefore, observed homogenization of larval bacterial communities at zoea stage could be explained by a growing influence of the rearing environment on the larval microbiota.

As for the water samples, high proportions of *Cytophagales* and *Bdellovibrionales* were associated with the larvae at zoea stage (from D2 to D5). However, unlike the water samples, abundances of *Caulobacterales* also increased during that rearing period. Members of this bacterial order have previously been identified as enriched in healthy *P. vannamei* and *P. stylirostris* larvae (Giraud et al. 2022, Yan et al. 2020). After the zoea stage, during the mysis stage (from D6 to D8), proportions of *Cellvibrionales* largely increased in the larvae samples. *Cellvibrionales* members have previously been highlighted in the gut microbiota of healthy *P. vannamei* adults that had been fed with probiotic *Bacillus* strains and challenged with *Vibrio* infection (Amoah et al. 2020). Therefore, *Caulobacterales* and *Cellvibrionales* may also be important for maintaining larval health during shrimp rearing.

### Identifying bacterial biomarkers in the water samples

Expected bacterial strains such as *Cognatishimia, Aestuariicoccus, Marivita, Owenweeksia, Alcanivorax, Litoricola, Aestuariibacter*, and *Alteromonas* were highlighted by the LEfSe analysis of the water samples (Fig. 5A). They are common marine bacteria and/or have already been highlighted in shrimp farming (Caporaso et al. 2011, Feng et al. 2018, Wang et al. 2020b, Giraud et al. 2021, Heyse et al. 2021, Lavoie et al. 2021, Wirth and Whitman 2018). More interestingly, *Nautella*, which were highlighted on D2 in the water samples (Fig. 5A), have been found associated with diseased *P. vannamei* larvae and were suggested as potential biomarkers of shrimp health status (Zheng et al. 2017, Callac et al. 2023, Zheng et al. 2016). However, they have also been identified in healthy shrimp larvae at zoea and mysis stage as well as in the intestinal microbiota of healthy adult organisms (Wang et al. 2020b, Restrepo et al. 2021). In this study, we have also highlighted the *Nautella* genus in the rearing water of healthy shrimp larvae (Fig. 5A). Thus, under the considered rearing conditions, presence of these microorganisms in shrimp rearing may not necessarily be a sign of upcoming mortalities. However, they may be opportunistic pathogens if shrimp health status deteriorates. In the same way, *Nioella*, which were enriched on D5 in the rearing water (Fig. 5A), have been identified as potential biomarkers of *P. stylirostris* larval mortalities and in adult *P. vannamei* affected by the white feces syndrome, where they were suggested to be part of a polymicrobial pathogen infection (Callac et al. 2023, Lu et al. 2020). As for *Nautella, Nioella* may be opportunistic pathogens that could lead to a microbial dysbiosis under specific conditions (Petersen and Round 2014). On the contrary, *Ruegeria* and *Leisingera*, which were predominant in the water samples on D5 and D9 (Fig. 5A) as well as in the larvae samples on D5 (Fig. 6A), have been reported as potential probiotics for shrimp and fish larval rearing (Amoah et al. 2020, Nyholm 2020, Callac et al. 2023) as they both exhibit antagonist activity against members of the *Vibrio* genus (Porsby et al. 2008, Gromek et al. 2016). In this study, *Ruegeria* and *Leisingera* were identified in healthy *P. stylirostris* larvae and in their rearing environment at different rearing periods suggesting that they could play a defensive role in the surrounding water and in the larvae by limiting proliferation of opportunistic pathogens or r-strategist microorganisms at key moments during the rearing process. Other bacterial strains considered to be beneficial microorganisms were highlighted during the rearing process in the water samples. Indeed, members of the *AEGEAN 169 marine group*, and belonging to the previously mentioned *Rhodospirillales* order, were enriched on D0 (Fig. 5A). This marine group has already been suggested to form the core microbiota of the eggs and the nauplii of *P. stylirostris* (Giraud et al. 2021). We previously suggested that *Rhodospirillales* could be a potential source of probiotic strains in the rearing water and further on suggest that members of the *AEGEAN 169 marine group* could be beneficial to shrimp larval rearing. However, this group is very diverse and further metagenomic studies will be necessary to identify precise microbial genera (Cram et al. 2015, Reintjes et al. 2019). Further on, *Pseudoalteromonas* were identified on D5 and D9. They are able to produce antibacterial compounds and have been identified as potential probiotic lineages for larval rearing of *P. stylirostris*. The NC201 strain is currently tested at the Saint Vincent experimental hatchery (Boulouparis, New Caledonia) (Pham et al. 2014, Sorieul et al. 2018, 2019). Interestingly, *Pseudoalteromonas* members have also been identified in the surrounding environment of *P. stylirostris* larvae subjected to high mortalities and were hypothesized to restrain microbial dysbiosis of the rearing water in order to prevent the development of opportunistic pathogens (Callac et al. 2022). *Saprospira, Lewinella*, and *Phaeodactylibacter* were enriched from D3 to D8 (Fig. 5A) in the rearing water. *Saprospira* were found in the eggs of the vent shrimp *Rimicaris exoculata* (Methou et al. 2019), while *Lewinella* have been identified in the water and the intestines of *P. vannamei* adults (Hou et al. 2018). *Phaeodactylibacter* were previously highlighted as significantly more abundant in mysis larvae of *P. stylirostris* as well as during the larval rearing of *P. vannamei*, where they originated from algal feed (Callac et al. 2024, Heyse et al. 2021). During our experiment, no algae were fed to the larvae. However, *Phaeodactylibacter* were abundant in the fourth type of microparticles (F4) given on D7 (Fig. 5B). As water samples were daily collected before the first feeding of the day, this could explain why *Phaeodactylibacter* were enriched in the rearing water on the following day (D8). Nevertheless, *Saprospira, Lewinella*, and *Phaeodactylibacter* belong to the *Chitinophagales* order and are, as previously discussed, capable of degrading the chitin composing the cuticle of the larvae. These bacterial strains could limit chitin accumulation in the rearing water occurring during the zoea transformation. The LEfSe also highlighted *Fabibacter*, affiliated to the *Cytophagales* order, in the rearing water on D4, during the zoea stage (Fig. 5A). *Fabibacter* are known to degrade complex organic molecules and have also been found associated with the cuticle of crustaceans (Reichenbach 1992), which could confirm our previous hypothesis that abundance of *Cytophagales* members increased due to larval molting and chitin release in the rearing water during zoea stage. As for *Saprospira, Lewinella*, and *Phaeodactylibacter, Fabibacter* may also restrict chitin concentrations in the larval environment at zoea stage. At the same time, *OM27 clade* members, which are *Bdellovibrio* and like organisms (BALOs), were enriched in the water samples on D5 (Fig. 5A) (Snyder et al. 2002, Bratanis et al. 2020). We have previously suggested that *Bdellovibrionales* could be a potential source of beneficial microbial strains for shrimp larval rearing and further on suggest that members of the *OM27 clade* could be of interest. However, as for the *AEGEAN 169 marine group*, the *OM27 clade* is very diverse, and further analysis will be necessary to identify more accurate taxa (Orsi et al. 2016).

### Identifying bacterial biomarkers in the larvae samples

In the egg samples, the LEfSe highlighted an ASV affiliated to the *Rhodobacteraceae* family and to the *Vibrio* genus (Fig. 6A), which is consistent with what has been previously highlighted. The *Rhodobacteraceae* family was suggested as potentially essential to shrimp larval rearing and as a source of potential probiotic taxa (Wang et al. 2020a), which strengthens the theory that they form the core microbiota of healthy shrimp larvae. ASVs affiliated to the *Rhodobacterales* order were highlighted at Z2, Z3, and M1-M2 stages (D4, D5, and D7): *Pseudooceanolicola, Leisingera*, and *Roseobacter*. To our knowledge, this study is the first to identify *Pseudooceanolicola* in larvae of a marine organism and further studies will be necessary to understand their role in *P. stylirostris* larvae. On the other hand, as previously mentioned, *Leisingera* have been considered as potential probiotics because of their ability to produce indigoidine, a compound demonstrating antimicrobial activity against *Vibrio* (Gromek et al. 2016). In the same way, *Roseobacter* have also been hypothesized to be probiotic microorganisms in marine animals as they produce a diverse range of secondary metabolites (Buchan et al. 2005, Porsby et al. 2008). The larvae LEfSe also highlighted various genera affiliated to the *Alteromonadales* in the larvae samples. Indeed, *Aestuariibacter, Alteromonas*, and *Thalassotalea* were highlighted at Z1, Z3, M1, and M3-PL stages (D2, D5, D6, and D9) (Fig. 6A). They have been previously identified as members of the core microbiota of the eggs and the nauplii of *P. stylirsotris* (Giraud et al. 2021). In the same way, *Pseudoalteromonas*, which were enriched in the larvae samples during the mysis/post-larvae transitioning phase (D9) (Fig. 6A), were found in the rearing water, the eggs, the nauplii, and the breeders of *P. stylirsotris* (Giraud et al. 2022). Thus, our experiment strengthens the hypothesis that *Aestuariibacter, Alteromonas, Thalassotalea*, and *Pseudoalteromonas* belong to the core bacteriota of shrimp larvae. From D3 to D6, *Cytophagales, Caulobacterales*, and *Bdellovibrionales* peeked in the larval microbiota (Fig. 4F). This was supported by the LEfSe, which highlighted *Fabibacter* and *Hyphomonas* on D4 (Z2 stage) as well as *OM27 clade* on D6 (Z3-M1 stages) (Fig. 6A). We have already discussed the implication of *Fabibater* and *OM27 clade* in shrimp larval rearing. The *Hyphomonadaceae* family has been identified in shrimp larvae and more specifically in the nauplii of the Pacific blue shrimp, where they were suggested to originate from the rare or inactive microbiota of the eggs and the rearing water (Giraud et al. 2022). As low abundances of *Hyphomonas* were also identified in the eggs and in the reservoir of the hatching tanks (Fig. 6B), our results reinforce this hypothesis.

### Correlations between environmental parameters and bacterial communities

Interestingly, all bacterial taxa highlighted by the LEfSe in the water and the larvae samples were found in the reservoir waters of the hatching and the rearing tanks as well as in the eggs, as shown by the constructed scatterplots (Figs 5B and 6B). The abundance of all taxa increased throughout the rearing days and the larval stages until reaching their maximum. This indicated that microorganisms present since the beginning of the experiment found more and more favorable conditions to their development throughout the rearing process. Several bacterial lineages identified as potential probiotics were highlighted during the experiment (Figs 5A and 6A), and their abundance varied greatly throughout the larval rearing (Figs 5B and 6B). However, all identified potential probiotics were found in the reservoir waters of the hatching and the rearing tanks suggesting that all these favorable microorganisms were present since the beginning of the rearing and originated from the natural LS (Figs 5B and 6B). Previous studies have already shown this early influence and, taken together (Giraud et al. 2021, Callac et al. 2022, 2023, 2024), this could lead to rethink the classic use of probiotics in aquaculture (i.e. a unique bacterial strain regularly inoculated throughout the rearing process) (Balcázar et al. 2006, Dawood et al. 2019) and lead to a more sustainable aquaculture by using a mix of various probiotic stains or by managing bacterial communities naturally found in the rearing environment. Indeed, our results suggest that several potential beneficial microbial strains with different functions are involved throughout the rearing at key moments of the larval development. Further analyses will be necessary to confirm the beneficial role of the highlighted microbial biomarkers, to better understand cellular and molecular mechanisms involved, and to establish better microbial management during shrimp larval rearing. In this context, we analyzed the correlations existing between the highlighted bacterial biomarkers and the physicochemical parameters to understand how the prokaryotic communities in the water and the larvae samples could be influenced.

More than half of the ASVs highlighted by the LEfSe of the water samples were correlated with at least one parameter related to organic matter concentration (Fig. 7A). This showed that the progressive eutrophication of the surrounding environment could influence the bacterial diversity of the rearing water. This was expected as eutrophication was proved to alter bacterioplankton assemblies in culture ponds of adult *P. vannamei* (Yang et al. 2018) and in the rearing water of *P. vannamei* larvae (Heyse et al. 2021). More generally, with growing impacts of human activities, such as agricultural activities and pollutant discharge, on fresh and seawater ecosystems, several studies have highlighted the influence of nitrogen and phosphorus enrichments on microbial communities (Qin et al. 2013, Liu et al. 2020). Eutrophication has also been hypothesized to increase bacterial growth and biomass, leading to an enhanced consumption of dissolved oxygen and, furthermore, to an enlarged CO_2_ concentration in the water causing pH to decrease (Smith and Schindler 2009). During our experiment, we indeed observed that pH values declined. However, as intensive bubbling of the rearing water was implemented in order to maintain oxygen levels, pH decrease was limited. Nevertheless, as pH variations and eutrophication are linked, it was not surprising that the ASVs positively correlated with organic matter concentrations were also negatively correlated with pH and *vice versa*. As for organic matter concentrations and pH, temperature is also known to strongly influence microbial compositions in all ecosystems (Aragno 1981). Interestingly, a study focusing on the microbiota associated with the intestines of adult *P. vannamei* as well as with their rearing water and sediments showed that pH, temperature, and total nitrogen most influenced the microbial communities of all samples (Huang et al. 2018). The authors showed that pH was the environmental factor most influencing the microorganisms in the water, while temperature had the biggest impacts on the microbial communities in the shrimps and in the sediment suggesting that environmental factors not only impacted the microorganisms associated to the water but also the microbiota of the animals. This was also highlighted by our findings, as several ASVs enriched in the larvae samples were correlated with the physicochemical parameters (Fig. 7B). As for the water samples, few ASVs were correlated with temperature indicating that this factor was potentially not the one most influencing the microbial communities associated with the larvae contrary to what Huang *et al*. have shown (Huang et al. 2018). Larvae-associated ASVs were strongly correlated with eutrophication parameters. In natural freshwater ecosystems, eutrophication of the water due to anthropogenic activities was proved to affect the microbial diversity of the skin microbiota of the wild gudgeon *Gobio occitanie*, therefore influencing the epibiota of the animals (Côte et al. 2022). Furthermore, the gastrointestinal microbiota of several fish species is known to be influenced by several environmental factors (Nayak 2010). These results suggest that the epibiota and the endobiota of *P. stylirostris* larvae could be influenced by changing environmental conditions throughout the rearing process.

Surprisingly, during zoea stage, less correlations were highlighted between bacterial communities in water and larvae samples suggesting that other unconsidered parameters might have come into play. In *P. vannamei* larvae, differential microbial lineage distributions were evidenced between nauplii and zoea stages as larval microbial colonization shifted from dispersal to host selection processes after the mouth opening (Wang et al. 2020a). More generally, a meta-analysis conducted on several freshwater and marine shrimp species showed that environmental factors influenced the microbiota of the animals but that the developmental stage was also an important factor shaping these microbial communities (Cornejo-Granados et al. 2018). Furthermore, many factors are involved when considering the evolution of host microbiota. Indeed, many biotic and abiotic factors, such as rearing conditions, diet, vertical transmission, and antibiotic addition, are known to influence symbiotic microbial communities (Nayak 2010, Cornejo-Granados et al. 2018). As a consequence, it is currently recognized that microbial community assemblies in aquaculture systems result from deterministic and stochastic processes (De Schryver and Vadstein 2014, Vadstein et al. 2018). Indeed, many deterministic processes, also called non-random and niche-based mechanisms, such as species traits, interspecies interactions (competition, predation, mutualisms. etc.), and environmental conditions are known to influence microorganisms in the environment (Zhou and Ning 2017). However, many studies focusing on the microbiota of marine larvae and their environment have found that other mechanisms could be involved and that deterministic processes were not sufficient to explain observed microbial patterns (Bakke et al. 2015, Giatsis et al. 2015, Heyse et al. 2021). Therefore, stochastic processes have been suggested to be involved in the assembly of microbial communities. Assuming that all species are ecologically functionally equivalent, these ecological mechanisms, ranging from probabilistic dispersal, random speciation or extinction, and ecological drift, are considered to generate microbial diversity patterns identical to those randomly generated (Zhou and Ning 2017). It is important to note that some environmental factors may not have been considered during our experiment explaining why few correlations were evidenced during the zoea stage in the water and the larvae samples. However, it is undeniable that stochastic processes may also be involved. In zebrafish, deterministic processes influence the establishment of the microbiota, while stochastic processes further shape the commensal gut microorganisms during the development (Yan et al. 2012). Thus, the same alternation between stochastic and deterministic processes may also explain the bacterial assembly of the Pacific blue shrimp larvae and their rearing water.

## Conclusion

**Figure 8.**
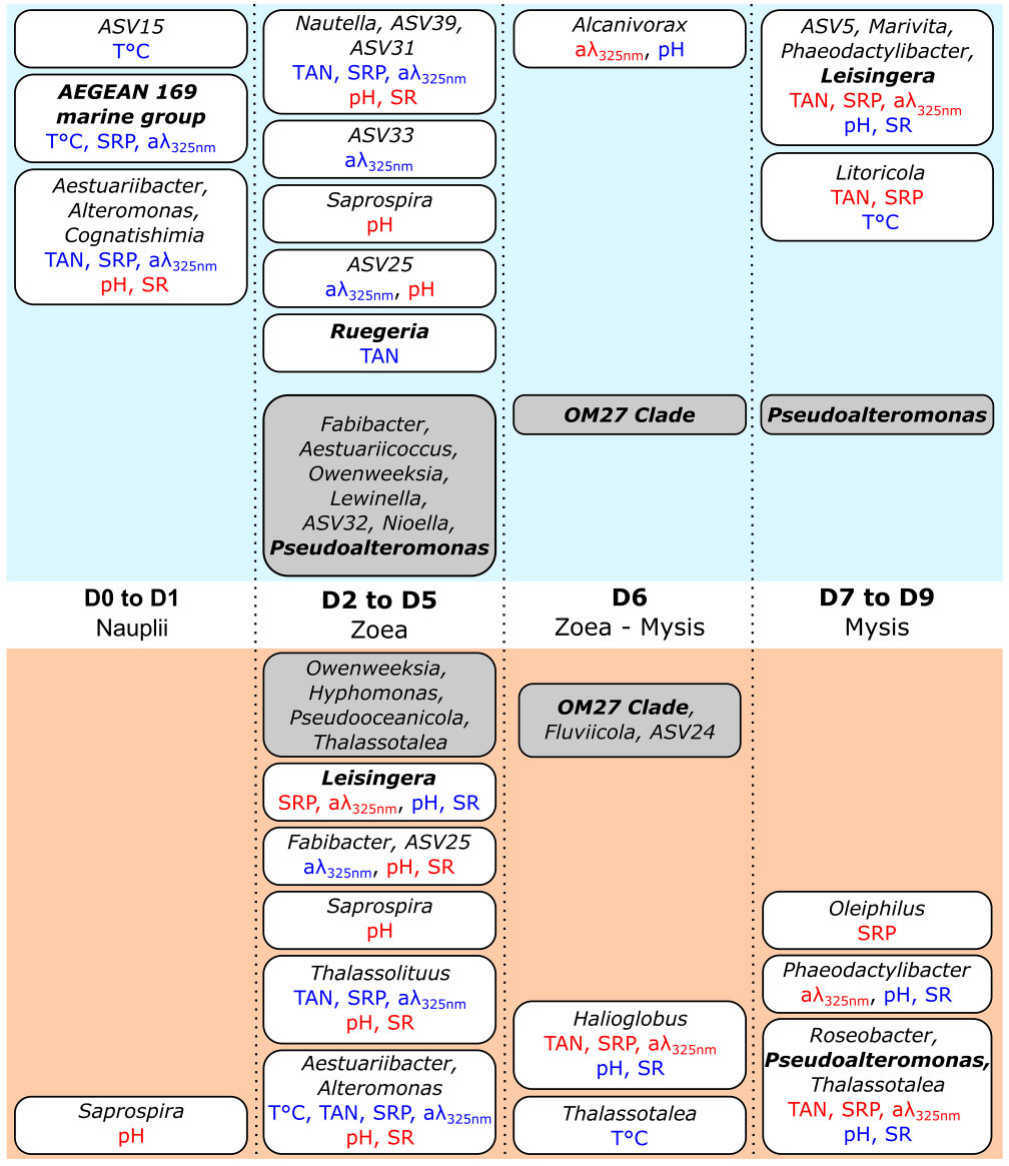
Enriched microbial lineages and their correlation with environmental factors. Enriched microbial lineages (up to the genus when possible) evidenced by the LEfSe in the water samples (in blue) and in the larvae samples (in orange). Environmental factors significantly correlated with these microbial lineages are noted in blue (negative correlations) or in red (positive correlations). Temperature (T°C), Total Ammonia Nitrogen (TAN), SRP, CDOM (aλ_325 nm_), pH, and SR were considered. Microbial lineages noted in gray boxes were not correlated with the considered environmental factors. Taxa in bold represent potential probiotic strains.

In conclusion, we were able to characterize the bacteriota associated with healthy *P. stylirostris* larvae and their rearing environment. We characterized the temporal evolutions of the physicochemical parameters of the rearing water as well as the prokaryotic communities associated with the larvae and their surrounding environment (Fig. 8). We showed a global eutrophication of the surrounding water implying drastic environmental changes between the beginning and the end of the rearing process with important shifts occurring during the zoea stage. Bacterial communities associated with the water and the larvae also strongly changed throughout the experiment according to the rearing day and the larval developmental stage. However, our results confirm that members of the *Alteromonadales, Rhodobacterales, Flavobacteriales, Oceanospirillales*, and *Vibrionales* bacterial orders could belong to the core microbiota of shrimp larvae. As these bacterial lineages were also found in the rearing water throughout the experiment, we hypothesize that these essential microorganisms may be acquired by the larvae from the rearing environment. Furthermore, several microbial variations were also observed and we identified, in the water and the larvae samples, several potential probiotic bacterial taxa affiliated to the *AEGEAN 169 marine group* and the *OM27 clade* as well as to *Ruegeria, Leisingera, Pseudoalteromonas*, and *Roseobacter*. As the abundance of these microorganisms varied throughout the experiment, we suggest that several beneficial bacterial strains could be involved during shrimp larval rearing, each playing a key role at a very specific time, leading to rethink probiotic use in aquaculture. Finally, we evidenced several correlations between the most abundant ASVs and the physicochemical parameters of the rearing water, which could confirm that deterministic processes are involved in the assembly of bacterial communities during the larval rearing of *P. stylirostris*. However, as few correlations were evidenced during the zoea stage, stochastic mechanisms may also come into play. Overall, important bacterial modifications occurred in the water and the larvae during this larval stage suggesting that the zoea phase is crucial during shrimp larval development.

## Supplementary Material

fiae156_Supplemental_Files

## Data Availability

The datasets generated and analysed during the current study are available in the NCBI SRA repository (BioProject PRJNA736535, SRA SRR21099695 to SRR21099837).
