# Supplementary material for: Environmental factors and potential probiotic lineages shape the active prokaryotic communities associated with healthy Penaeus stylirostris larvae and their rearing water
Source: FEMS Microbiol Ecol. 2024 Nov 19;100(12):fiae156. doi: 10.1093/femsec/fiae156 (PMC11636268; doi:10.1093/femsec/fiae156)
Supplement: fiae156_Supplemental_Files [file fiae156_supplemental_files.zip › Supplementary_figures.docx]

| 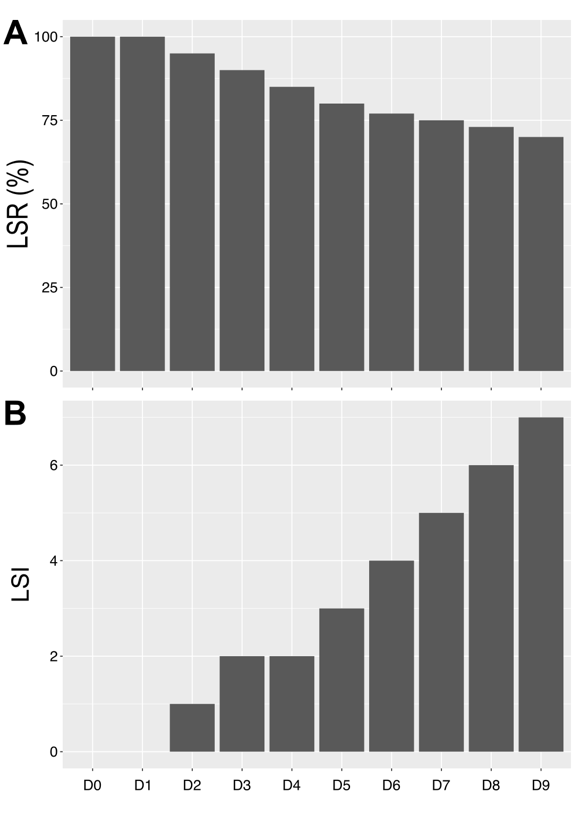 |
| --- |
| ***Supplementary figure 1 – Reference values for larval survival rate (LSR) and larval stage index (LSI).***  *A) Larval survival rate (LSR) and B) Larval stage index (LSI) values expected for each rearing day. Rearing days are represented on the horizontal axis by the letter D followed by the considered rearing day (from 0 to 9).* |

| 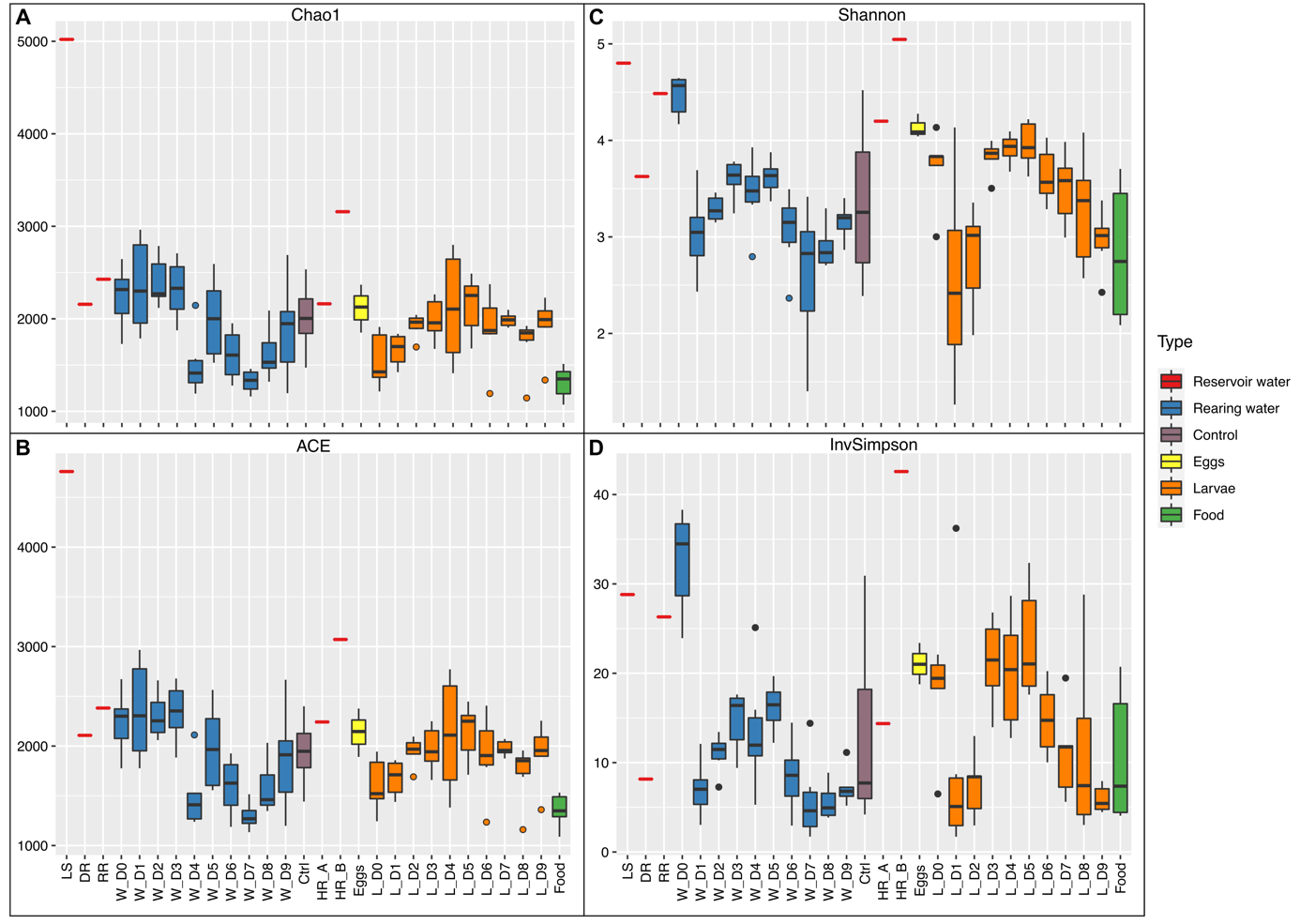 |
| --- |
| ***Supplementary figure 2 - α-diversity indices in all the samples.***  *(A) Chao1, (B) ACE, (C) Shannon and (D) Inverse Simpson (InvSimpson) indexes in all the samples. LS stands for lagoon seawater, DR for decantation reservoir, RR for rearing reservoir, Ctrl for control and HR for hatching reservoir. All the water samples are designated by a W while all the larvae samples are designated by an L. The rearing days are represented by the letter D followed by the considered rearing day (from 0 to 9).* |

| 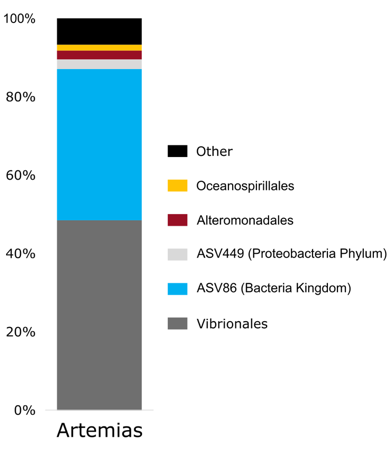 |
| --- |
| ***Supplementary figure 3 – Microbial composition of the Artemias sp. larvae.***  *Top 5 bacterial orders (when possible) with a relative abundance higher than 1% associated with the Artemias sp. larvae.* |
